# Retraction: Trapping mosquitoes using milk products as odour baits in western Kenya

**DOI:** 10.1186/1756-3305-3-78

**Published:** 2010-08-25

**Authors:** Eunice A Owino

**Affiliations:** 1 School of biological sciences, The University of Nairobi, P.O Box 30197,G.P.O, Nairobi, Kenya

## Retraction

This article [1] has been retracted by the Editor because there are unresolved issues relating to authorship and content.

## References

[B1] Owino EA Trapping mosquitoes using milk products as odour baits in western Kenya Parasit Vectors 2010 3 1 55 10.1186/1756-3305-3-55 20573278 PMC2931484

